# QL^4^MDR: a GraphQL query language for ISO 11179-based metadata repositories

**DOI:** 10.1186/s12911-019-0794-z

**Published:** 2019-03-18

**Authors:** H. Ulrich, J. Kern, D. Tas, A. K. Kock-Schoppenhauer, F. Ückert, J. Ingenerf, M. Lablans

**Affiliations:** 1 IT Center for Clinical Research, University of Lübeck, Lübeck, Germany; 2 Federated Information Systems, German Cancer Research Center, Heidelberg, Germany; 3 Medical Informatics in Translational Oncology, German Cancer Research Center, Heidelberg, Germany; 4 Institute of Medical Informatics, University of Lübeck, Lübeck, Germany

**Keywords:** Metadata repository, Interoperability, GraphQL, HL7 FHIR

## Abstract

**Background:**

Heterogeneous healthcare instance data can hardly be integrated without harmonizing its schema-level metadata. Many medical research projects and organizations use metadata repositories to edit, store and reuse data elements. However, existing metadata repositories differ regarding software implementation and have shortcomings when it comes to exchanging metadata. This work aims to define a uniform interface with a technical interlingua between the different MDR implementations in order to enable and facilitate the exchange of metadata, to query over distributed systems and to promote cooperation. To design a unified interface for multiple existing MDRs, a standardized data model must be agreed on. The ISO 11179 is an international standard for the representation of metadata, and since most MDR systems claim to be at least partially compliant, it is suitable for defining an interface thereupon. Therefore, each repository must be able to define which parts can be served and the interface must be able to handle highly linked data. GraphQL is a data access layer and defines query techniques designed to navigate easily through complex data structures.

**Results:**

We propose QL^4^MDR, an ISO 11179-3 compatible GraphQL query language. The GraphQL schema for QL^4^MDR is derived from the ISO 11179 standard and defines objects, fields, queries and mutation types. Entry points within the schema define the path through the graph to enable search functionalities, but also the exchange is promoted by mutation types, which allow creating, updating and deleting of metadata. QL^4^MDR is the foundation for the uniform interface, which is implemented in a modern web-based interface prototype.

**Conclusions:**

We have introduced a uniform query interface for metadata repositories combining the ISO 11179 standard for metadata repositories and the GraphQL query language. A reference implementation based on the existing Samply.MDR was implemented. The interface facilitates access to metadata, enables better interaction with metadata as well as a basis for connecting existing repositories. We invite other ISO 11179-based metadata repositories to take this approach into account.

## Background

Heterogeneity of healthcare data from different sources is a well-known obstacle limiting data integration and analytics. If the same facts are expressed in various ways, understanding and exchanging data becomes a demanding process that ties up resources in the form of data specialists and is both labor-intensive and error-prone [1].

**Fig. 1:**
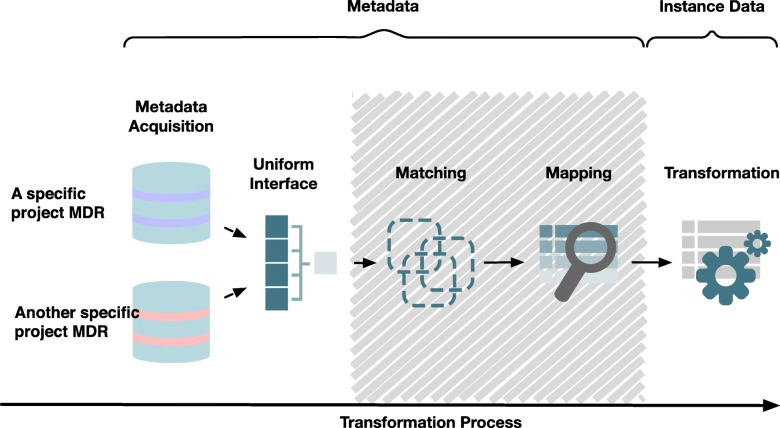
Using metadata to support the integration of healthcare instance data. The process consists of the four stages: the metadata acquisition stage with a uniform interface enables to reuse of information which is stored in project-specific MDRs. The matching stage aligns the metadata and identifies potential correspondences. The mapping stage creates transformation rules, which are used in the transformation stage. The first three stages only process metadata, whereas the last transformation stage includes healthcare instance data

As a remedy, the unambiguous interpretation and, thus, integration of such “instance data” can be facilitated by describing their variety and characteristics using “metadata”. If curated and semantically annotated, metadata is instrumental in data integration [2]. For example, metadata can be used for validation and transformation of instance data: Having harmonized metadata at the schema level, matchings and mappings between different metadata sets can be used to generate the transformation of instance data, as conceptually shown in Fig. 1. It has been shown that such processing rules can serve to integrate and exchange healthcare instance data [3].

Many projects and organizations in the field of medical informatics research already utilize metadata repositories (MDR) to store, edit, use and reuse metadata. As a result, a multitude of MDR implementations have emerged, each one featuring its own web interface, e.g. the Common Data Element Browser from the National Institute of Health [4], the US Health Information Knowledgebase, the Samply.MDR [5] and the METeOR of the Australian Institute of Health and Welfare [6]. While mapping between data elements within one MDR is a well-researched topic, the exchange between several MDRs – a requirement for the exchange and integration process across consortia – has been much less studied. This shall be the focus of this study, represented on the left side in Fig. 1. Fortunately, most MDR systems claim to be partly conformant to the metadata standard ISO 11179, so that in principle metadata can be exchanged between MDRs [4, 7–10]. However, while ISO 11179-3 defines a metamodel and basic attributes for describing metadata, it does not provide an implementation. After studying the several systems mentioned above, we discovered that some systems either provide no query endpoint at all, or the existing interfaces are rather deprecated. Existing metadata exchange standards are not focused on the ISO 11179 standard, are proprietary and rigid due to their design and technologies [11]. The semantically enhanced metadata is therefore unavailable due to technical or syntactical heterogeneity. In summary, before we can exploit metadata from several MDRs for data integration, we face a problem of metadata integration.

We propose a uniform interface to access multiple MDRs as long as they follow a specific metadata standard. The idea of a uniform interface (of clinical systems) has a prominent example through HL7 Fast Health Interoperability Resources (FHIR) [12]. Standardized exchange formats are provided equipped with modern tooling like JSON, ATOM and REST. Although, the standard is disadvantageous if deeply structured resources are to be processed. Since metadata is predominantly a deeply nested information, it is urgently dependent on implementing effective access to real MDR systems on a technical level.

### The ISO 11179 standard for metadata repositories

In order to design a uniform interface suitable for several existing MDRs, a standard data model needs to be agreed on. The ISO/IEC standard 11179 is commonly used for the modelling of metadata, corresponding repositories and registries [13]. The standard defines a core model in order to harmonize the formal representation of metadata. This core model is divided into two layers: the representational and the conceptual layer. The representational layer defines the key concept *Data Element* as a single information element and a *Value Domain* describing the datatypes and their value ranges. The conceptual layer sorts *Data Element* in concept groups to describe their semantical similarity. In addition to the core model, the standard defines various entities to capture the information corresponding to the metadata. As an objective, the interface must be able to query a highly linked data model.

### Fast health interoperability resources

A uniform interface is a common way to overcome the problem of heterogeneity in data exchange. A significant example is the Fast Health Interoperability Resources standard, the newest member of the HL7 standards family [12]. FHIR defines information components, called resources, and a standardized way to retrieve and manipulate these components. The FHIR resource *DataElement* and the ISO 11179 profile, defined for representing metadata in FHIR version DSTU2, were the base for a functional MDR prototype [14]. With FHIR version STU3, however, the *DataElement* resource has been marked deprecated, and a suitable successor has not been defined, yet. In particular, FHIR developers state that REST interfaces are not a suitable communication approach for the complex, nested queries as required in exchange of ISO 11179-3 information [15].

### GraphQL

GraphQL, initially developed by Facebook, is a query language especially suited for highly linked data models [16] used by GitHub, Twitter or the German railway company Deutsche Bahn [17, 18]. The FHIR standard itself introduced GraphQL as a query alternative to REST APIs [19]. Technically, GraphQL functions as a database abstraction layer providing a single API endpoint both for queries and mutations. The provided information objects are defined in a *schema*, which has an expressive coverage, supports inheritance, interfaces, custom types and attribute constraints such as non-nullable entries. Creating a GraphQL schema requires to define:

- *Objects and Fields* to define information representation
- *Queries* to define how object types can be queried, including filtering and
- *Mutations* to enable input types for information capturing and manipulation.

Providing a GraphQL endpoint based on a given schema is achieved by implementing data fetchers and resource resolvers collecting the enquired resources and providing them in the defined format. Apart from the interface specification, GraphQL supports introspection based on the underlying schema, so the interface information is machine-readable available to simplify interaction with clients to generate communication libraries automatically [20]. It also provides reference implementation and software libraries in various programming languages, like JavaScript, Erlang, C# and Java [16].

### Implementation

We used the GraphQL reference library *graphql-java* [20] to derive the QL^4^MDR API and its documentation from the defined schema. As a next step, we implemented the API in a widely used open-source ISO 11179-based metadata repository, Samply.MDR [21]. We created the necessary data fetchers using the underlying Samply.MDR database access layer that ensures backwards compatibility across MDR versions and allows the use of the existing access control based on OpenID Connect [22]. As an optimization, we implemented resource resolvers to reduce the necessary connections to the database via lazy-loading, e.g. fetching a namespace including each data element with the corresponding value domains without producing a large number of database queries.

## Results

Having reviewed the ISO 11179-3 core model, we propose a compatible GraphQL schema, a GraphQL-based API QL^4^MDR and a prototypical implementation of a modern web-based interface.

### Definition of an ISO 11179-compatible GraphQL schema

We derived the GraphQL schema for QL^4^MDR from the ISO 11179 standard. Particularly the third part describing the core model, was considered. Of the 26 entities described in the core model, the QL^4^MDR schema consists of the following: a) Object types with corresponding fields, b) Query and c) Mutation types.

### Objects & Fields

The ISO 11179-3 core model is represented in four Object types: *Data Element*, *Value Domain*, *Data Element Concept* and *Conceptual Domain*. The standard also comprises *Namespace* and the customizable *Slots* as structures for the identification of metadata. Also, all required Objects related to the previous six types are included in the QL^4^MDR schema, resulting in 13 Object types.

ISO 11179-3 further specifies these basic Object types by attributes. We translated these attributes into GraphQL fields, which can be used to filter and constrain the query. To enhance filter functionality, Object types with less than two attributes are included in related Objects as fields. For example, the ISO 11179 *Property Class* results in the string representation Property related to the *Data Element Concept.*

### Query

GraphQL queries start at an *entry point* and traverse through the data graph. QL^4^MDR provides six entry points: *Data Element* as the central information item, *Value Domain*, *Concepts* and *Conceptual Domain, Namespaces* and *Slot*. Each entry point provides a particular set of filters to specify the enquired information, e.g. all concepts regarding P*erson* and its *mass*. Since slots can contain custom information about each data element, they allow additional parameters for better querying.

**Fig. 2:**
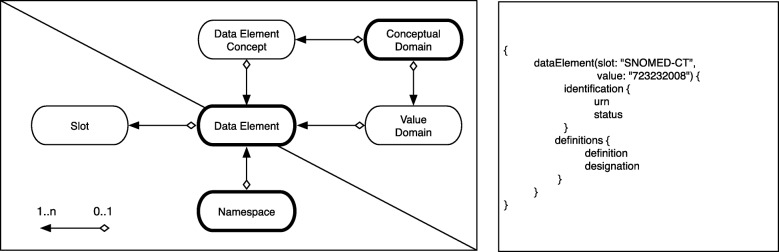
The six defined entry points, separated into the identified metadata (lower part) and the formal description of the metadata (upper part). The three bold entities are suitable entry points for mutations. The right box shows an example query to request all data Data Elements containing a Slot with the name “SNOMED-CT” and the value “723,232,008” (average blood pressure). The query defines the representation of the response: each corresponding Data Element shall be returned with its identification and its definitions

The QL^4^MDR data graph has a defined direction, which we derived from the cardinality described in the ISO 11179-3 – represented with directed lines in Fig. 2. QL^4^MDR queries should be formulated in a way traversing the graph along the defined directions.

### Mutation

Of the six available entry points for querying, we selected three as valid starting points for mutations: *Namespace*, *Conceptual Domain* and the pivotal *Data Element*. This selection ensures two important guarantees: first, each entity can be created, modified or deleted as there is a guaranteed path. Second, it is impossible to define cyclical mutations.

## Discussion

The proposed interface follows two major design decisions, which result in advantages with regards to MDR interoperability: choosing GraphQL rather than RESTful or a service-oriented interface and basing the QL^4^MDR on the ISO 11179-3 standard rather than a proprietary implementation.

### GraphQL vs. traditional interfaces

**Fig. 3:**
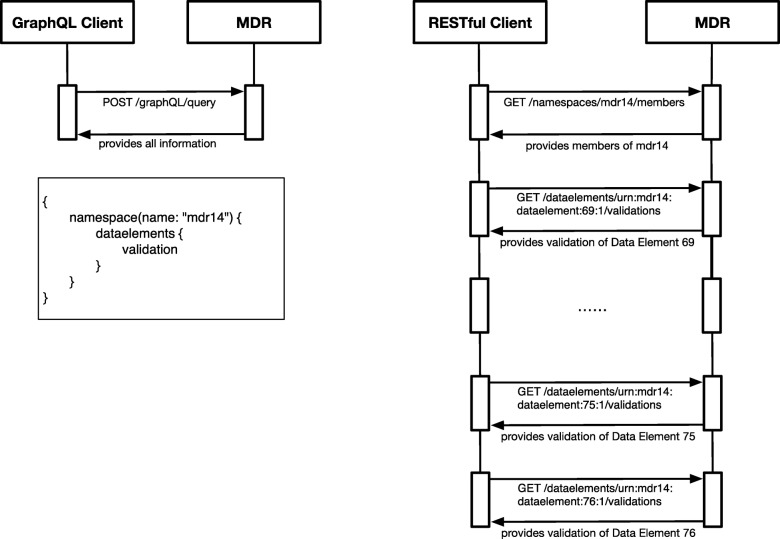
This sequence diagram shows the required messages between the GraphQL client (left) including the used query (box), the RESTful client (right) and the MDR server to receive the validation rules of each data element in a specific namespace. The GraphQL client needs only one query shown in the box, whereas the message amount of the RESTful client depends on the number on data elements associated with the chosen Namespace

GraphQL can be regarded as a variation of the widely used RESTful design pattern but differs in specific characteristic and yields both advantages and limitations: As a GraphQL-based API, QL^4^MDR can answer even complex questions navigating across the various entities of the ISO 11179 standard, thus reducing the required number of queries. In other words, the RESTful or service-oriented interfaces need substantially more requests to provide the same information. The number of queries against a RESTful interface depends on the number of inquired data elements. For example, consider an electronic data capture solution requesting validation rules for all data elements present in a given namespace, as shown in Fig. 3**.** Additionally, a RESTful client receives redundant information as it is forced to query data elements with all properties and has to discard those that are of no further benefit [16]. The RESTful interface could implement tailored routes, but it is infeasible in the comparison of benefit from costs due to maintenance.

In GraphQL, clients can define the desired response format with each query, which future-proofs the interface for new client requirements. This shifts the workload back from the client to the server to be compliant with a larger number of client implementations but yields technical limitations compared to REST. On the one hand, the deeply nested queries are well-suited to the highly networked metadata as they include more information and therefore reduce request roundtrips. On the other hand, they cause a higher load on the MDR databases. Even worse, GraphQL does not rely on standard HTTP mechanics and therefore does not profit from the well-matured caching mechanisms of modern web browsers and client libraries, which further amplifies the database load with repeated queries [23]. Facebook is aware of this shortcoming and provides a JavaScript library to overcome this obstacle [16].

Another advantage of GraphQL lies in the creation of meaningful documentation. In particular, GraphQL implementations like *graphql-java* can generate both human- and machine-readable documentation from the defined schema. The introspection feature allows not only users and developers to understand the interface more easily, but the machine-readable representation enables dynamic and loose coupling between server and clients [16], thus facilitating the federation of various, technically different ISO 11179-based MDRs. Previous standards like the *WS-MetadataExchange* [11] cannot stand that flexibility and loose coupling due to its heavyweight service-oriented architecture [24].

### Adherence to metadata standards instead of their implementations

QL^4^MDR is not tailored to a specific repository implementation but modelled strictly after the ISO 11179-3 standard. This approach yields both advantages and limitations.

On the one hand, adhering to ISO 11179-3 as the common metadata model ensures reusable queries that can be executed against various MDR implementations, as long as they follow ISO 11179-3 and implement QL^4^MDR. On the other hand, metadata management systems are sometimes customized for specific use cases and specifications, which go beyond what ISO 11179-3 defines. For instance, Samply.MDR implements the so-called *Data Element Group* to organize certain data elements. As this entity is not included in the standard, it obviously cannot be queried via QL^4^MDR. However, workarounds are possible: in this case, for example, *Data Element Groups* could be treated as complex data elements consisting of several data elements, a designation and a definition.

### Limitations

Designing a common interface is the first step on the way to a simple federation of heterogeneous MDRs via a uniform and standardized interface and therefore reusing metadata. An interface alone, however, cannot address common problems of handling of metadata in a distributed context, such as consolidation of datasets and/or the mediation between existing sets, matching and mapping of data elements and protection of intellectual property (study designs, etc.). Also, federating various MDR instances yields the usual problems of distributed information systems such as replication, consistency and duplicate detection, addressing and operational availability and versioning. QL^4^MDR is made for MDRs which are based on the 11,179–3, non-ISO-based systems are currently out of scope.

Lastly, one must consider that like any other interface, QL^4^MDR can offer only functionality or serve information available in the underlying MDR. In the case of ISO 11179, not all MDRs implement all components of the extensive standard. For example, although QL^4^MDR does cover the conceptual layer, it is unavailable in our reference implementation as it is not available in Samply.MDR. To some extent, such limitations can be mitigated: In our example, the additional semantic information can be stored in the optional slot of a data element. However, for the sake of interoperability across MDR implementations, we argue that compliance to the ISO 11179 standard is preferable to such workarounds.

## Conclusion

We have presented a uniform query interface for various implementations of metadata repositories. To ensure compatibility and sustainability, we did not invent new paradigms but reused existing standards, namely the widely used ISO 11179 standard for metadata registries and the GraphQL query language. We implemented a reference implementation based on the widely used Samply.MDR software, which is available under https://bitbucket.org/medicalinformatics/. QL^4^MDR could be integrated into other MDR implementations following the ISO 11179 metadata representation by implementing the required GraphQL data fetcher and the HTTP-based query endpoint. Once integrated into MDRs, QL^4^MDR can not only enable better interaction with a single metadata repository in a uniform and based on the ISO 11179-3 standardized manner. In addition, it serves as the foundation towards a federation of existing implementations and research networks’ instances. Thus, we invite authors of other ISO 11179-based metadata registries to consider this approach for implementation.

## Availability and requirements

The source-code are freely released in open source on Bitbucket.

**Project name**: e.g. Samply.MDR.GraphQL.

**Project home page**: e.g. https://bitbucket.org/medicalinformatics/samply.mdr.ql4mdr

**Operating system(s)**: Platform independent.

**Programming language**: Java.

**Other requirements**: Java 1.3.1 or higher, Tomcat 4.0 or higher.

**License**: GNU Affero General Public License.

**Any restrictions to use by non-academics**: no licence needed.

## Abbreviations

FHIR: Fast Health Interoperability Resources
MDR: Metadata Repositories

## References

[1] Khoumbati K, Themistocleous M, Irani Z. Integration Technology Adoption in Healthcare Organisations: A Case for Enterprise Application Integration. Proceedings of the 38th Annual Hawaii International Conference on System Sciences. 2005:9.

[2] Dugas M (2016). Design of case report forms based on a public metadata registry: re-use of data elements to improve compatibility of data. Trials.

[3] Aubrecht P, Kouba Z. Metadata Driven Data Transformation. In: ISAS-SCI (1). Citeseer; 2001. p. 332–336.

[4] Nadkarni PM, Brandt CA (2006). The common data elements for cancer research: remarks on functions and structure. Methods Inf Med.

[5] Kadioglu D, Weingardt P, Lablans M, Ückert F, Wagner TO. Samply. MDR–Ein Open-Source-Metadaten-Repository. German Medical Science GMS Publishing House. 2016.

[6] Australien Institute of Health and Welfare. METeOR home. http://meteor.aihw.gov.au/content/index.phtml/itemId/181162. Accessed 29 Jun 2018.

[7] Stausberg J, Löbe M, Verplancke P, Drepper J, Herre H, Löffler M (2009). Foundations of a metadata repository for databases of registers and trials. Stud Health Technol Inform.

[8] Ngouongo SM, Löbe M, Stausberg J (2013). The ISO/IEC 11179 norm for metadata registries: does it cover healthcare standards in empirical research?. J Biomed Inform.

[9] Richesson RL, Nadkarni P (2011). Data standards for clinical research data collection forms: current status and challenges. J Am Med Inform Assoc.

[10] Park YR, Yoon YJ, Kim HH, Kim JH (2013). Establishing semantic interoperability of biomedical metadata registries using extended semantic relationships. Stud Health Technol Inform.

[11] Ballinger K, Box D, Curbera F, Davanum S, Ferguson D, Graham S, et al. Web services metadata exchange (WS-MetadataExchange). OASIS draft. 2004.

[12] Benson T, Grieve G. Principles of Health Interoperability. Springer; 2016.

[13] ISO/IEEC 11179–3. Information Technology – Metadata Registries (MDR), Part 3: Registry Metamodel and Basic Attributes, Edition 3, see https://www.iso.org/standard/50340.html. 2013.

[14] Ulrich H, Kock A-K, Duhm-Harbeck P, Habermann JK, Ingenerf J (2016). Metadata repository for improved data sharing and reuse based on HL7 FHIR. Stud Health Technol Inform.

[15] Hay D. GraphQL | Hay on FHIR. https://fhirblog.com/2017/08/17/graphql/. Accessed 2 Jul 2018.

[16] Buna S. Learning GraphQL and relay: Packt Publishing Ltd; 2016.

[17] Facebook Inc. GraphQL: Users. http://graphql.org/users. Accessed 6 Jun 2018.

[18] DB Systel GmbH. API-Portal - 1BahnQL-Free. https://developer.deutschebahn.com/store/apis/info?name=1BahnQL-Free&version=v1&provider=DBOpenData. Accessed 6 Jun 2018.

[19] Health Level 7. Graphql - FHIR v3.4.0. http://build.fhir.org/graphql.html. Accessed 27 Jun 2018.

[20] Facebook Inc. GraphQL: A query language for APIs. http://graphql.org/. Accessed 27 Jun 2018.

[21] Kadioglu D, Breil B, Knell C, Lablans M, Mate S, Schlue D (2018). Samply.MDR - a metadata repository and its application in various research networks. Stud Health Technol Inform.

[22] Sakimura N, Bradley J, Jones M, de Medeiros B, Mortimore C. OpenID Connect Core 1.0 incorporating errata set 1. The OpenID Foundation, specification. 2014.

[23] Kern J, Tas D, Ulrich H, Schmidt EE, Ingenerf J, Ückert F, et al. A Method to use Metadata in legacy Web Applications: The Samply.MDR.Injector. Stud Health Technol Inform - In Press. 2018. 30147038

[24] Kumari S, Rath SK. Performance comparison of soap and rest based web services for enterprise application integration. In: Advances in Computing, Communications and Informatics (ICACCI), 2015 International Conference on. IEEE; 2015. p. 1656–1660.

